# Extensively Drug-Resistant Tuberculosis in Women, KwaZulu-Natal, South Africa

**DOI:** 10.3201/eid1710.110105

**Published:** 2011-10

**Authors:** Max R. O’Donnell, Jennifer Zelnick, Lise Werner, Iqbal Master, Marian Loveday, C. Robert Horsburgh, Nesri Padayatchi

**Affiliations:** Albert Einstein College of Medicine, Bronx, New York, USA (M.R. O’Donnell);; Centre for AIDS Programme of Research in South Africa, Durban, South Africa (M.R. O’Donnell, L. Werner, N. Padayatchi);; Touro College Graduate School of Social Work, New York, New York, USA (J. Zelnick);; King George V Hospital, Sydenham, South Africa (I. Master);; Medical Research Council, Cape Town, South Africa (M. Loveday);; Boston University School of Public Health, Boston, Massachusetts, USA (C.R. Horsburgh)

**Keywords:** Tuberculosis, HIV/AIDS, gender, epidemiology, extensively drug resistant tuberculosis, South Africa, tuberculosis and other mycobacteria, women, dispatch

## Abstract

To determine whether women in KwaZulu-Natal, South Africa, with drug-resistant tuberculosis (TB) were more likely than men to have extensively drug-resistant TB, we reviewed 4,514 adults admitted during 2003–2008 for drug-resistant TB. Female sex independently predicted extensively drug-resistant TB, even after we controlled for HIV infection. This association needs further study.

Tuberculosis (TB) remains a leading infectious cause of death worldwide (*1*), especially where HIV is endemic (*2*). In industrialized countries, outbreaks of drug-resistant TB (multidrug-resistant [MDR] and extensively drug-resistant [XDR] TB) have occurred predominantly among male patients (*1*). However, in South Africa, where TB and HIV are endemic, aggregate data suggest that a greater proportion of women than men with TB have MDR TB (*3*).

In South Africa, HIV infection is more prevalent among women than men. Nationally, women 25–29 years of age have the highest (32.7%) HIV prevalence; for men, prevalence peaks at age 30–34 years (25.8%) (*4*). KwaZulu-Natal Province, the epicenter of the HIV/AIDS epidemic in South Africa, has a high incidence of hospital admissions for MDR TB and XDR TB and >70% HIV co-infection among MDR TB patients (*5*). Since 2003, fueled by a generalized HIV epidemic, TB incidence in South Africa (*1*) and hospital admissions for MDR TB in KwaZulu-Natal have doubled (*5*). Observational studies of MDR TB and XDR TB in South Africa report higher proportions of female patients with MDR TB or XDR TB (*6*–*9*). We conducted this study to determine whether women in KwaZulu-Natal with drug-resistant TB were more likely than men to have XDR TB, even after we controlled for HIV and other factors.

## The Study

The study design has been described (*5*). Briefly, we retrospectively reviewed adult MDR TB and XDR TB patients admitted during 2003–2008 for treatment initiation to King George V Hospital (KGVH), a public TB-referral hospital in KwaZulu-Natal. Admitting physicians and staff collected data routinely. During the study period, KGVH was the only public hospital in the province authorized to initiate treatment for MDR TB and XDR TB, and all therapy was initiated on an inpatient basis.

All patients >18 years of age who had culture-confirmed MDR TB or XDR TB with standard drug susceptibility testing were included. Repeat admissions were excluded. MDR TB and XDR TB were defined according to standard definitions (*10*). Ethics review committees at the University of KwaZulu-Natal and Boston University (Boston, MA, USA) approved the study protocol.

We used Fisher exact or χ^2^ tests to compare categorical variables. Medians were compared by using the Wilcoxon-Mann-Whitney U test. Univariate and multivariate logistic regression models were used to estimate odds ratios (ORs) and 95% confidence intervals (CIs). Statistically significant variables or variables that caused >10% change in the univariate OR were included in the multivariate model. Interaction terms were assessed by using Wald tests, with p<0.2 considered significant. Test for trend was performed by using the Cochran-Armitage test. Analysis was performed with SAS version 9.3 software (SAS Institute, Inc., Cary, NC, USA).

A total of 4,941 patients with MDR TB or XDR TB from throughout KwaZulu-Natal were admitted for initiation of drug-resistant TB treatment to KGVH during the study period (Figure). Among 4,514 eligible patients with MDR TB or XDR TB, women were younger (median age [interquartile range] 32 [26–39] vs. 36 [30–44] years), more likely to be HIV infected (65% vs. 47%), and more likely than men to be receiving antiretroviral therapy (ART) (51% vs. 43%) (Table 1). Women with drug-resistant TB were more likely than men with drug-resistant TB to have XDR TB (p<0.0001).

**Figure:**
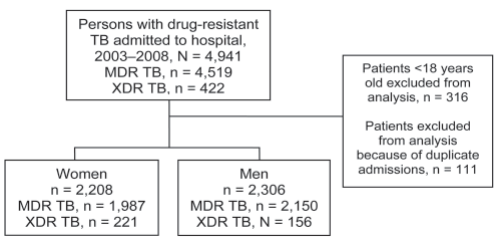
Flow diagram for patients with multidrug-resistant (MDR) and extensively drug-resistant (XDR) tuberculosis (TB) admitted to King George V Hospital, KwaZulu-Natal, South Africa, 2003–2008.

**Table 1:** Characteristics of 4,514 adults >18 years of age with MDR TB and XDR TB admitted to King George V Hospital, KwaZulu-Natal, South Africa, 2003–2008*

Characteristic†	No. (%) women, n = 2,208	No. (%) men, n = 2,306	p value
HIV status			
Positive	1,431 (64.8)	1,083 (47.0)	<0.0001
Negative	394 (17.8)	642 (27.8)	
Unknown	383 (17.3)	581 (25.2)	
HIV positive and ART			
Yes	731 (51.1)	465 (42.9)	<0.0001
No	700 (48.9)	618 (57.1)	
Health care worker			
Yes	180 (8.2)	51 (2.2)	<0.0001
No	2,028 (91.8)	2,255 (97.8)	
Previous treatment			
Yes	1,987 (95.4)	2,127 (94.4)	0.1305
No	96 (4.6)	127 (5.6)	
Year of hospital admission			
2003	188 (8.5)	278 (12.1)	0.0011‡
2004	195 (8.8)	234 (10.1)	
2005	272 (12.3)	283 (12.3)	
2006	358 (16.2)	350 (15.2)	
2007	590 (26.7)	593 (25.7)	
2008	605 (27.4)	568 (24.6)	
Type of TB			
MDR	1,987 (90.0)	2,150 (93.2)	<0.0001
XDR	221 (10.0)	156 (6.8)	

*MDR, multidrug-resistant; TB, tuberculosis; XDR, extensively drug-resistant; ART, antiretroviral therapy. †Median patient age (interquartile range): women, 32 y (26–39 y); men, 36 y (30–44 y); p<0.0001. ‡Cochran-Armitage test for trend.

On univariate analysis, HIV status, ART (among HIV-infected), female gender, previous TB treatment, and year of admission to KGVH were significantly associated with XDR TB (Table 2). On multivariate analysis, only female gender (OR 1.38, 95% CI 1.11–1.73), previous TB treatment (OR 2.16, 95% CI 1.09–4.28), and year of admission were independently associated with XDR TB. In the HIV strata, ART was not significantly associated with XDR TB after adjustment was made for confounding variables. Confounding of ART and XDR TB by year of admission among HIV co-infected persons most likely resulted from increased XDR TB case finding after a highly publicized XDR TB outbreak in 2006 and increased ART use after public health rollout of ART in KwaZulu-Natal in 2004. The interaction terms HIV × gender, gender × age (categorical), and health care worker (HCW) × gender were not significantly associated with XDR TB.

**Table 2:** Risk factors for XDR TB and MDR TB among adults >18 years of age admitted to King George V Hospital with drug-resistant TB, KwaZulu-Natal, South Africa, 2003–2008*

Risk factor	No. (%) with XDR TB, n = 377	No. (%) with MDR TB, n = 4,137	OR (95% CI)	
			Univariate analysis	Multivariate analysis
Sex				
F	221 (58.6)	1,987 (48.0)	1.53 (1.24–1.90)	1.38 (1.11–1.73)
M	156 (41.4)	2,150 (52.0)	Reference	Reference
Age, y†				
18–35	204 (54.1)	2,265 (54.7)	0.98 (0.79–1.20)	–
>36	173 (45.9)	1,872 (45.3)	Reference	
HIV status‡				
Positive	259 (68.7)	2,255 (54.7)	1.43 (1.10–1.87)	1.19 (0.90–1.56)
Negative	77 (20.4)	959 (23.2)	Reference	Reference
Unknown	41 (10.9)	923 (22.3)	0.55 (0.38–0.82)	0.69 (0.46–1.04)
Previous TB treatment				
Yes	359 (97.6)	3,755 (94.6)	2.27 (1.16–4.47)	2.16 (1.09–4.28)
No	9 (2.5)	215 (5.4)	Reference	Reference
HIV positive and ART‡				
Yes	149 (57.5)	1,047 (46.4)	1.56 (1.21–2.03)	–
No	110 (42.5)	1,208 (53.6)	Reference	
Year of admission				
2003	6 (1.6)	460 (11.1)	Reference	Reference
2004	5 (1.3)	424 (10.2)	0.90 (0.27–2.98)	0.96 (0.27–3.33)
2005	36 (9.5)	519 (12.5)	5.32 (2.22–12.74)	5.03 (1.94–12.99)
2006	78 (20.7)	630 (15.2)	9.49 (4.10–21.97)	9.26 (3.71–23.13)
2007	137 (36.3)	1,046 (25.3)	10.04 (4.40–22.91)	9.14 (3.70–22.60)
2008	115 (30.5)	1,058 (25.6)	8.33 (3.64–19.07)	7.45 (3.00–18.50)

*XDR, extensively drug-resistant; TB, tuberculosis; MDR, multidrug-resistant; OR, odds ratio; CI, confidence interval; ART, antiretroviral therapy. †Median age (interquartile range): MDR TB patients 34 y (28–42 y); XDR TB patients, 35 y (29–42 y). ‡Univariate analysis restricted to HIV-positive persons only. ART use excluded from multivariate analysis because persons receiving ART must be HIV infected.

Most (59%) patients admitted with XDR TB were women, which did not change significantly during the study period (test for trend p = 0.68). For MDR TB, the data showed increasingly more female MDR TB patients admitted over the study period (p<0.001) (Table A1).

## Conclusions

Our major finding was that women admitted with drug-resistant TB to KGVH were 38% more likely than men to have XDR TB. This association remained significant after adjustment for potential confounding variables, including HIV status. Temporal analysis showed persistently more women with XDR TB and increasing proportions of women with MDR TB during the study period. Together these data support the notion that the epidemic of drug-resistant TB predominantly affects women in KwaZulu-Natal.

Supporting context comes from observational studies of drug-resistant TB in South Africa. In South African studies, higher percentages of XDR TB (50%–56%) than MDR TB patients (43%–53%) are women (*6*–*9*). However, studies from low-prevalence HIV settings report fewer women with drug-resistant TB. In a study in the United States, few patients with MDR TB (36%) or XDR TB (38%) were female (*11*). Similarly, in cohorts from Latvia, Peru, and Russia, lower percentages of patients with MDR TB (17%–40%) and XDR TB (29%–35%) were female (*12*–*14*). Gender differences in drug-resistant TB in areas of HIV endemicity and low prevalence suggest a possible effect of the AIDS epidemic on prevalence of drug-resistant TB in women.

Our study has several limitations. We lacked details about hospital admission and factors associated with referral. Women with XDR TB might have been preferentially referred compared with men with XDR TB, women with MDR TB, or both. We lacked data on HIV factors, such as CD4 T-cell counts, ART adherence, and viral load. Similarly, we lacked data on previous TB treatment, medications, adherence, and outcome. Finally, as a hospital-based retrospective study, factors associated with survival to hospital admission, decisions to seek care, and referral patterns may introduce bias.

Potential causes of the association between female gender and XDR TB are not known. HIV-related factors could explain the association of XDR TB and female gender. For example, women with drug-resistant TB may be more adherent to ART, leading to improved survival, and therefore increased time for XDR TB to develop. Factors associated with secondary development of XDR TB, such as TB medication adherence or previous MDR TB treatment, could explain the association between XDR TB and female gender (*15*). Factors associated with exposure to drug-resistant TB strains, including location and duration of exposure, could explain the association of XDR TB and female gender. For example, women are more likely to participate in formal and informal care work, with potential exposure to drug-resistant TB strains, and therefore primary XDR TB might be more likely to develop (*5*).

These results may have major policy implications for TB control in KwaZulu-Natal and South Africa. Because women are more likely to have XDR TB in KwaZulu-Natal, efforts should be made to develop gender-sensitive interventions to improve diagnosis, treatment, and prevention for drug-resistant TB and HIV. Decentralization of drug-resistant TB treatment may better accommodate women in conjunction with their work, family, and child-rearing responsibilities. Further studies are needed to confirm the magnitude and determinants of the association between female gender and XDR TB.

## Appendix

**Table A1:** Trends in hospital admissions for MDR TB and XDR TB and HIV prevalence among adults >18 years admitted to King George V Hospital, KwaZulu-Natal, South Africa*

	MDR TB patients		XDR TB patients		HIV prevalence†	
Year of hospital admission	Male, no. (%), n = 2,150	Female, no. (%), n = 1,987	Male, no. (%), n = 155	Female, no. (%), n = 222	Male, no. (%), n = 1,083	Female, no. (%), n = 1,431
2003	275 (59.8)	185 (40.2)	3 (50.0)	3 (50.0)	80 (51)	64 (74)
2004	231 (54.5)	193 (45.5)	2 (40.0)	3 (60.0)	82 (55)	94 (72)
2005	268 (51.6)	251 (48.4)	15 (41.7)	21 (58.3)	126 (63)	159 (75)
2006	321 (51.0)	309 (49.0)	29 (37.2)	49 (62.3)	143 (61)	221 (76)
2007	532 (50.9)	514 (49.1)	61 (44.5)	76 (55.5)	331 (66)	432 (81)
2008	523 (49.4)	535 (50.61)	45 (39.1)	70 (60.9)	321 (66)	461 (81)
Cochran-Armitage test for trend, p value	<0.001		0.68		<0.001	0.003

*MDR TB, multidrug-resistant tuberculosis; XDR TB, extensively drug-resistant tuberculosis.

†HIV prevalence among MDR TB and XDR TB patients with known HIV test results.
